# Does relapse matter? Insights from new data in schizophrenia

**DOI:** 10.1192/bjp.2025.10470

**Published:** 2025-11-10

**Authors:** Oliver Howes, Maria Kapi, Bernard R. Bukala

**Affiliations:** 1 Institute of Psychiatry, Psychology and Neuroscience, King’s College London, UK

## Abstract

It is often assumed that relapse leads to poor long-term outcomes, but new data question this for symptoms, social function, quality of life and, possibly, employment. We consider this together with other impacts, risks and costs and how individual circumstances all influence decisions about antipsychotic maintenance treatment to prevent relapse.

Substantial evidence shows that maintenance treatment with antipsychotic medicine reduces the risk of relapse in schizophrenia. This includes the results from more than 90 randomised controlled trials(1) plus large pharmaco-epidemiological studies(2) showing that the risk of relapse is roughly halved in people continuing on antipsychotic treatment relative to those who discontinue antipsychotics. The number needed to treat (NNT) to prevent one relapse is around 3,(1) which compares favourably with many other interventions in medicine.

However, does preventing relapse matter? This is an important question because antipsychotic maintenance treatment is not a zero-sum game. Some antipsychotic treatments have been shown in randomised controlled trials to induce cardiometabolic side-effects.(3) Tardive dyskinesia remains a risk even with newer dopamine D2/3 receptor blocking drugs, and is irreversible in some people.(4) Sedation, sexual impairment, motor dysfunction, prolactin elevation and other side-effects affect quality of life.(5) D2/3 receptor blockade may also induce secondary negative symptoms and impair functioning.(4) It is not surprising, then, that many patients want to discontinue maintenance antipsychotic treatment. A further consideration is the health service costs of maintenance treatment: the direct cost of the medicine, the clinician and pharmacist time monitoring treatment, and the costs of investigations to monitor side-effects and interventions to address them. This all diverts resources from other uses. On top of this, there is the psychosocial impact on the patient and their carers of maintenance treatment: the implicit message that they remain unwell, and in need of care, and the time they spend on taking treatment.

These factors highlight how important it is to understand whether relapse matters. There are two aspects to this: preventing relapse itself and preventing the consequences of relapse. There is substantial evidence that duration of untreated psychosis is associated with poorer long-term outcomes.(6) This could suggest that relapse of psychosis is also associated with lasting effects, including higher long term symptom severity, and poorer long term social and occupational function.(7) However, it is surprising how few studies have investigated the long-term impact of relapse, particularly given the number of studies that have investigated whether antipsychotic treatment reduces the risk of relapse. Moreover, most of the studies have used convenience samples to compare outcomes in patients who have relapsed with those who have not. Consequently, a major issue with many of these studies is sampling bias: effects could be driven by baseline differences in the severity of illness, for example. In this context, a recent randomised trial provides an important addition to the evidence base on the long-term outcomes following relapse (Moncrieff et al BJPsych In Press 2025).

The report by Moncrieff et al (2025) is a secondary analysis of data from the RADAR trial, which recruited over 250 chronically ill patients taking antipsychotic treatment for recurrent psychosis or schizophrenia. Patients were randomised to either gradual, flexible antipsychotic dose reduction with the aim of stopping treatment (the discontinuation group) or continuing on the current dose of antipsychotic treatment (the maintenance group). Social functioning was the primary outcome measure but they also collected data on symptom severity, quality of life and employment status. They compared change in these measures from baseline to 24 months follow-up.

Importantly, a large proportion of patients (190/253, 75%) completed the 24-month follow-up. 82 patients experienced at least one relapse during the follow-up period. The study found no significant difference in any of the outcome measures at 24-months between patients who relapsed and those who had not. There was a significantly higher risk of relapse in the discontinuation group relative to the maintenance group. Nevertheless, the results of the long-term outcomes were essentially the same after adjusting for whether patients were randomised to maintenance or discontinuation group. Moreover, there was no significant change in any of the measures when the baseline data were compared to the follow-up data in those patients who had experienced a relapse.

Clinicians and experts by experience (see box 1) often report that a patient’s illness does not respond as well to treatment following a relapse as it did before relapse. Real world evidence also indicates that the dose of antipsychotic increases following each relapse.(2) However, one of the most striking findings of the Moncrieff et al study is that symptom severity at 24 months was not significantly different from baseline levels in those whose illness had relapsed. This is not compatible with the hypothesis that relapse is associated with poorer subsequent response to treatment on average, although information on antipsychotic dose at follow-up is not reported so it is unclear if this changed following relapse relative to baseline – in future studies it would be important to establish the effect of relapse on the antipsychotic treatment required, which as discussed comes with significant costs. Nevertheless, overall, these data indicate that relapse is not associated with long-term effects on social function, symptom severity, or quality of life.

It is important to consider several factors in the study design and patient population to know which patients these results are most applicable to. Importantly, the authors note that the majority of participants in the RADAR trial were “male, white, single and unemployed”, so must be generalised with caution to other patient populations. Patients entering the trial consented to randomisation and follow-up, and people were excluded if they were legally required to take antipsychotic treatment. We can speculate that this is likely to have enriched the sample for people who were relatively well engaged with mental health services and who, consequently, would re-engage with treatment quickly following a relapse to maximise the chance of recovery. This may limit the applicability of the findings to patients who do not engage well with services. The patients entering the trial also showed relatively low symptom severity at baseline. Whilst we don’t have any information on the severity of symptoms prior to entering the study, this suggests that their illnesses had responded well to prior treatment. Similarly, we have limited information on the relapse history of patients prior to entry to the trial, but given the baseline symptom ratings were low, it seems they had recovered well to their previous relapse. The findings of this study may, thus, be less applicable to patients where their illness has shown only a partial response to treatment.

Another important characteristic of the RADAR study population is the chronicity of their condition, and that patients had mostly had multiple previous relapses (on average 3 previous hospital admissions). Evidence from other studies suggests that antipsychotic treatment response is lower following the second (and subsequent relapses) relative to the first relapse, suggesting that preventing the second relapse is particularly important.(2) Therefore, it would valuable for RADAR and future similar studies to include subgroup analyses comparing long-term outcomes in patients with one prior relapse to those who have had two or more prior relapses.

Around 25% of the sample were in employment, education or training at baseline. This is fairly representative for people with schizophreniform disorders in the United Kingdom, but means the study was probably underpowered to detect differences in employment status between those who experienced a relapse relative to those who did not. For example, to detect a 10% difference in employment rates at p<0.05 (two-tailed) requires a sample size of 490 with 80% power, when the baseline employment rate is 25% (two-sample test of independent proportions using G*Power 3.1). It is interesting to note that in numerical terms, the proportion of people in employment, education or training had dropped more in the relapse group than the group who had not relapsed. Thus, the finding of no significant difference between groups in this outcome measure should be treated with caution. It is also important to note that relapse may have other long-term consequences that were not measured in this study, such as loss of friends and self-esteem, and effects on career progression (box 1).

Notwithstanding the issues discussed above, the finding that there is no evidence that relapse is associated with long-term effects across multiple measures is a reassuring outcome for patients and clinicians. Perhaps relapse is not so terrible after all? However, patients and clinicians also need to consider the effects of relapse itself. Acute deterioration in mental state can present serious risks to self and others, and damage social relationships (box 1). Relapse also increases health service costs, particularly if it leads to admission to hospital (which occurred in 25% of participants in the antipsychotic reduction group in the RADAR trial).(8)

In conclusion, whilst it is clear that antipsychotic maintenance treatment reduces the risk of relapse in schizophrenia, the decision to take maintenance treatment depends on multiple factors, including the impact of side-effects, the direct consequences of relapse as well as its long-term effects. The new data from Moncrieff et al (2025) add important evidence on key elements of the latter in patients with chronic illness, low risk and low levels of symptoms. There are also outstanding issues, including whether employment was affected, the health economic effects, and whether findings will generalise to other patient groups, particularly those who are more unwell prior to discontinuation. This highlights the need for more studies into the long-term risks and benefits of antipsychotic maintenance treatment. Finally, it is important to recognise that the large number of factors that may influence the risk-benefit analysis and the importance an individual gives to them make the decision to continue maintenance treatment a complex and highly personal choice. Decision aids may help by supporting patients and clinicians to identify what is important to the individual.(9) Clinicians and patients should also recognise that the balance may change with changing circumstances and values, meaning the decision needs to be both individualised and re-visited. One size does not fit all, all the time!

## Box1 The impact of relapse: a personal perspective

I have lived with schizophrenia for the last 20 years and have had six relapses during this time. After each relapse, my antipsychotic medicine has not been as effective as it was before the relapse and the dose has had to be increased. Every relapse has also had great effect on my self-esteem and it takes considerable time to return back to normal and smile from my heart again. For me, relapse is a devastating experience each time. An important long-term consequence of my relapses has been the loss of some friends who couldn’t accept me when I had psychosis. This has narrowed my friendship circle. Not all relapses have had the same long-term consequences on my life. Ten years ago, I had a relapse and lost my employment, and professional registration. I recovered and got a different job three months later. However, my professional registration was only restored nine years later, which affected my career progression as I couldn’t apply for jobs that required a licence to practice as a doctor. I consider this an important consequence. In contrast, I had another relapse almost 2 years ago and my employer treated me with great care and respect, provided me with the time to recover and I didn't lose my job. In my experience, the impact of relapse varies, and it is very much dependent on your circumstances at the time, your place in your patient journey, and how well you can manage a relapse.

Dr Maria Kapi, expert by lived experience and training
